# Tetrodotoxins Secretion and Voltage-Gated Sodium Channel Adaptation in the Ribbon Worm *Kulikovia alborostrata* (Takakura, 1898) (Nemertea)

**DOI:** 10.3390/toxins13090606

**Published:** 2021-08-29

**Authors:** Anna E. Vlasenko, Vasiliy G. Kuznetsov, Grigorii V. Malykin, Alexandra O. Pereverzeva, Peter V. Velansky, Konstantin V. Yakovlev, Timur Yu. Magarlamov

**Affiliations:** A.V. Zhirmunsky National Scientific Center of Marine Biology, Far Eastern Branch, Russian Academy of Sciences, 690041 Vladivostok, Russia; avlasenko@imb.dvo.ru (A.E.V.); vkuznetsov@imb.dvo.ru (V.G.K.); gmalykin@imb.dvo.ru (G.V.M.); apereverzeva@imb.dvo.ru (A.O.P.); pvelansky@imb.dvo.ru (P.V.V.); kyakovlev@imb.dvo.ru (K.V.Y.)

**Keywords:** nemertea, ribbon worm, tetrodotoxin, TTX, tetrodotoxin analogues, TTX resistance, Nav channel mutation

## Abstract

Nemertea is a phylum of marine worms whose members bear various toxins, including tetrodotoxin (TTX) and its analogues. Despite the more than 30 years of studying TTXs in nemerteans, many questions regarding their functions and the mechanisms ensuring their accumulation and usage remain unclear. In the nemertean *Kulikovia alborostrata*, we studied TTX and 5,6,11-trideoxyTTX concentrations in body extracts and in released mucus, as well as various aspects of the TTX-positive-cell excretion system and voltage-gated sodium (Nav1) channel subtype 1 mutations contributing to the toxins’ accumulation. For TTX detection, an immunohistological study with an anti-TTX antibody and HPLC-MS/MS were conducted. For Nav1 mutation searching, PCR amplification with specific primers, followed by Sanger sequencing, was used. The investigation revealed that, in response to an external stimulus, subepidermal TTX-positive cells released secretions actively to the body surface. The post-release toxin recovery in these cells was low for TTX and high for 5,6,11-trideoxyTTX in captivity. According to the data obtained, there is low probability of the targeted usage of TTX as a repellent, and targeted 5,6,11-trideoxyTTX secretion by TTX-bearing nemerteans was suggested as a possibility. The Sanger sequencing revealed identical sequences of the P-loop regions of Nav1 domains I–IV in all 17 studied individuals. Mutations comprising amino acid substitutions, probably contributing to nemertean channel resistance to TTX, were shown.

## 1. Introduction

Nemertea is a phylum of marine worms, consisting of more than 1300 species, most of which are active predators [1]. Nemerteans are subdivided into three phylogenetic groups—pilidiophora, paleo-, and hoplonemerteans. Representatives of all three phylogenetic groups bear various toxins [2], including tetrodotoxin (TTX), a non-proteinaceous low-molecular-weight toxin [3].

Despite the more than 30 years of studying TTX in nemerteans, many questions regarding its functions and the mechanisms ensuring its accumulation and usage remain unclear [2]. Most studies have focused on toxin detection in nemerteans [4,5,6,7,8] and the search for toxin sources [9,10,11], while a small number of studies included physiological experiments concerning the transportation and function of TTX and its analogues (TTXs) in ribbon worms. The physiological experiments dedicated to the study of the secretion of the highly toxic nemertean *Cephalothrix simula* (=*C. linearis*) showed the presence of TTX in mucus secreted upon stimulation of the animal [12,13]. Ali et al. [12] showed that the amount of TTX in the secretions of *C. simula* decreased with each subsequent stimulation, depleting the toxin reserves in the glands after 3–6 days of daily sampling. The amount of TTX remaining in the body was much higher than the total amount of the secreted toxin, which corresponds to the study, indicating that TTX is mainly localized in the intestine of the nemertean consequently cannot be secreted [5]. For many TTX-secreting animals, the function of the toxin as a predator deterrent was suggested. The realization of this function in ribbon worms supposes the recovery of TTX in secreting cells through migration from the tissues of the internal environment. In the current research, using 17 specimens of the ribbon worm *Kulikovia alborostrata*, we studied the dynamics of TTX concentration in the secretion produced at different time intervals, and four specimens were used for the investigation of toxin localization at different stages of the excretion process.

To accumulate TTXs and specifically use them as antipredator defense or for prey immobilization during hunting, animals should have molecular mechanisms ensuring resistance to the toxin. The resistance mechanisms known for some TTX-bearing animals represent mutations in TTX targets, such as voltage-gated sodium (Nav) channels in the region of the selective filter [14]. In the current research, for the first time, a search for the genetic mutations leading to a decrease in the affinity of the Nav1 channel to TTX in nemerteans was performed.

## 2. Results

### 2.1. TTXs

**Figure 1:**
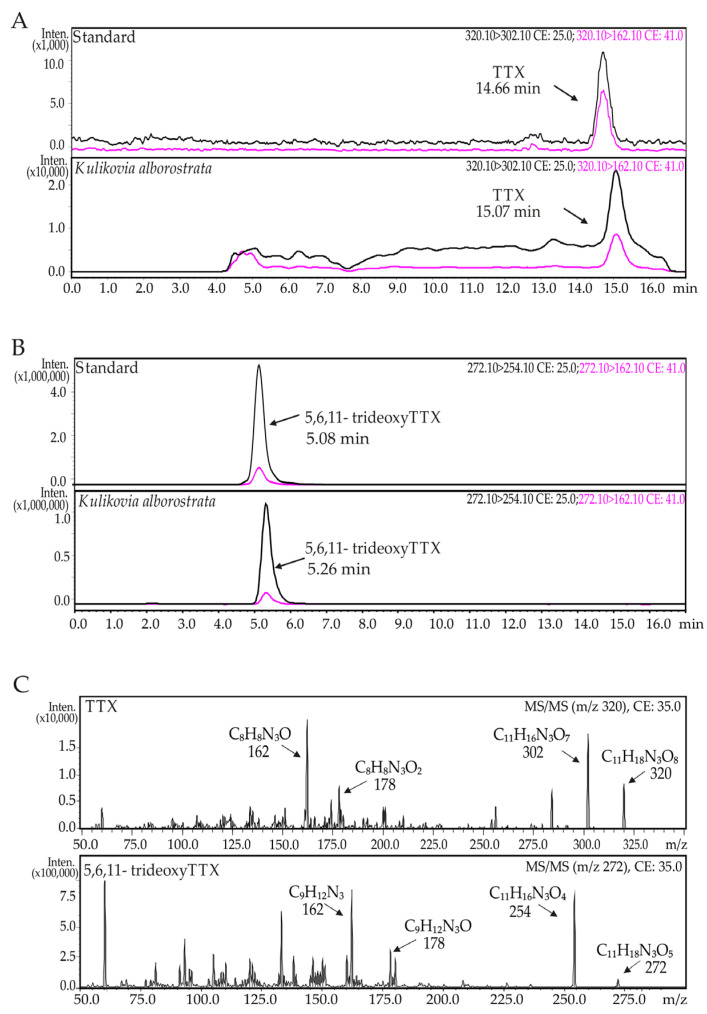
(**A**,**B**) High-performance liquid chromatography—tandem mass spectrometry (HPLC-MS/MS) chromatograms of tetrodotoxin (TTX) and 5,6,11-trideoxyTTX from *Kulikovia alborostrata* extracts; (**C**) MS/MS spectra of (TTX) and 5,6,11-trideoxyTTX from *K. alborostrata* extracts.

The TTX content in the secretions and body extracts of nemertean *K. alborostrata* was studied with high-performance liquid chromatography (HPLC) coupled with tandem mass spectrometry (MS/MS). Two toxins—namely TTX and 5,6,11-trideoxyTTX—were detected in both sample types (Figure 1).

For complete removal of the secreted toxins from the secretion structures, a control three-day stimulation of all 17 animals for secretion collection was carried out, with one stimulation per day. On the first day, 6 out of 17 specimens were shown to possess TTXs in secretions; on Day 2, this number fell to 13 specimens; and on Day 3, to 4 specimens. At the same time, the concentration of TTX in the studied samples did not exceed 0.556 ng/g of body weight, and the concentration of 5,6,11-trideoxyTTX varied from 0.144 (Worm 13) to 0.675 (Worm 1) ng/g of body weight on the first day, from 0.156 (Worm 3) to 6.244 (Worm 4) ng/g of body weight on the second day, and from 0.093 (Worm 15) to 0.287 (Worm 13) ng/g of body weight on the third day. The ribbon worms were kept for 30 days, and, after this time, measurements of the TTX concentrations in their secretions showed an increase in 16 out of 17 worms, while the TTX concentration in the remaining specimen was higher than the limit of quantification (LoQ) (0.6 ng/mL of the extract) (Worm 4); therefore, it could be clearly quantified. In six samples, the concentration of 5,6,11-trideoxyTTX did not exceed the LoQ; in the rest, it varied from 0.068 (Worm 15) to 0.862 (Worm 7) ng/g of body weight. In the Day 210 day (seventh month), all 17 experimental specimens showed the presence of TTX in the excreted mucus, whereas 5,6,11-trideoxyTTX was not detected. At this time, the concentration of TTX exceeded the LoQ only in Worm 9, while in the rest, it was lower. After the last secretion collection in the Day 210, the bodies of all studied specimens were analyzed for the presence of TTXs. It was found that the body extracts of the studied ribbon worms contained only TTX; in 11 specimens, the toxin concentration did not exceed the LoQ, and in six specimens, its concentration was 0.146–1.286 ng/g of body weight (Table 1).

### 2.2. Nav1 Channel

As a result of Sanger sequencing, four P-loop regions of domains I–IV constituting the selectivity filter of the Nav1 channel were obtained. The lengths of the sequenced regions of domains I, II, III, and IV were 280, 388, 313, and 268 bp, respectively. The processing of data from all 17 *K. alborostrata* specimens showed that the amino acid sequences of the obtained regions were identical for all examined individuals.

**Figure 2:**
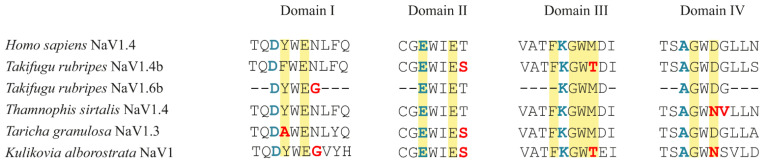
Sequence alignment of P-loop regions of Nav channels domains I–IV of *Homo sapiens* (TTX-sensitive channel), TTX-resistant animals (*Takifugu rubripes*, *Thamnophis sirtalis*, *Taricha granulosa*), and *Kulikovia alborostrata,* studied herein. The four key amino acids constituting the selectivity region—aspartate-glutamate-lysine-alanine (DEKA) motif are marked in blue. Amino acid substitutions associated with TTX channel resistance are marked in red. Amino acids involved in TTX binding and coordination are marked in yellow according to Shen and colleagues [17]. Accession numbers or references: *H. sapiens* Nav1.4: P35499; *T. rubripes* Nav1.4b: Q2XVR6; *T. rubripes* Nav1.6b: [16]; *T. granulosa* Nav1.3: A0A6G9W273; *T. sirtalis* Nav1.4: A0A1W5T2B2.

As a result, in domain I, no substitutions were revealed in positions directly binding TTX (Figure 2) according to the literature [15,16,17]; however, a rare mutation was found—the substitution of asparagine (Asn) with glycine, also shown for the Nav1.6b channel in toxic *Takifugu rubripes* [16]. Shen and colleagues [17] assumed that, although Asn is not directly involved in TTX binding, a substitution in this position can reduce local electronegativity and decrease the channel’s affinity for TTX. In the region of the domain II P-loop (Figure 2) of *K. alborostrata*, threonine (Thr) substitution with serine was observed, which was previously shown in TTX-resistant *Taricha granulosa* Nav1.3 [16] and *T. rubripes* Nav1.4b [18] (Figure 2). In domain III, a mutation that led to the substitution of methionine and isoleucine with Thr was identified, which was also observed in other TTX-bearing organisms (Figure 2). It has been shown that this substitution reduces the TTX sensitivity of the Nav channel 15-fold [16] as these amino acids directly interact with the TTX oxygen anion [17]. In domain IV of TTX-sensitive Nav channels (*Homo sapiens* Nav1.4), tryptophan is followed by aspartic acid, while some toxin-containing species, including *K. alborostrata*, have Asn in this position. This configuration was found in Nav1.4 in some populations of the TTX-resistant garter snake *Thamnophis sirtalis* and increased channel resistance 300-fold [19].

### 2.3. Immunohistochemical Study

**Figure 3:**
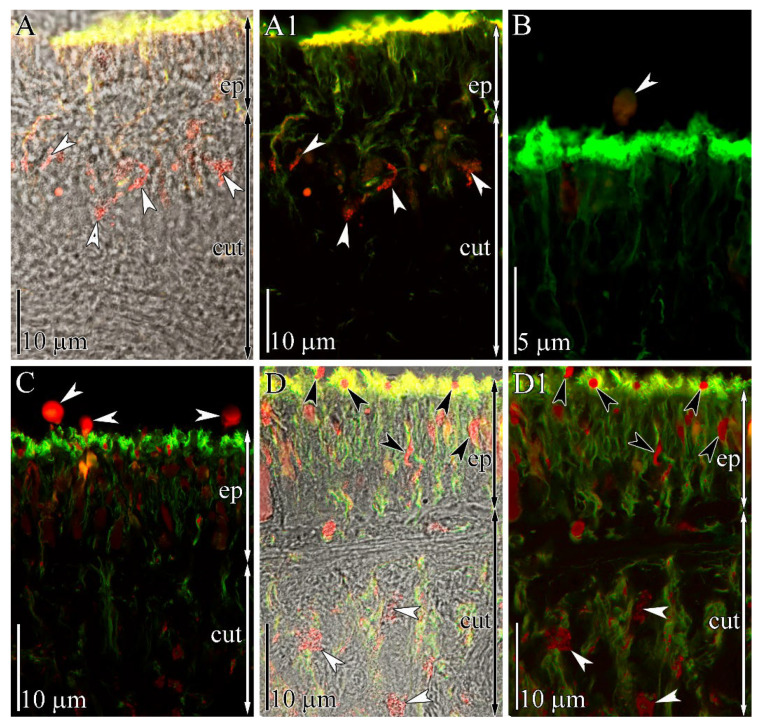
Confocal laser scanning micrographs (Z-projections) of transverse sections of the body wall of the intact (**A**,**B**) and stimulated (**C**,**D**) specimens of *Kulikovia alborostrata*. Red: tetrodotoxin-like immunoreactivity; green: cilia and cytoskeleton, acetylated tubulin immunoreactivity. (**A**,**A1**) Panoramic view showing subepidermal gland cells with toxin-positive granules (arrowheads). (**B**) TTX-positive spherical secretion (arrowhead) above the apical surface of the epidermis. (**C**) Panoramic view showing TTX-positive spherical secretions (arrowheads) above the apical surface of the epidermis. (**D**,**D1**) Panoramic view of integument showing subepidermal gland cells with TTX-positive granules (white arrowheads) and their epidermal extensions with TTX-positive granules (black arrowheads). Legends: cut, cutis; ep, epithelium.

The integument of *K. alborostrata* belongs to the heteronemertean type and consists of two sublayers: the epidermis and the subepidermal layer (cutis) [20]. TTX localization in the tissue and gland cells of intact specimens of *K. alborostrata* was previously examined on the light optical and electron microscopy levels [21]. According to this report, the integument contains only one type of TTX-positive gland cell (named subepidermal bacillary gland cells type I). The bodies of these cells are localized in the cutis and form long ducts passing through the epidermis and opening out on its surface. In this study, we also revealed TTX-positive gland cells in the cutis and their ducts in the epidermis (Figure 3A,A1). The epidermal ducts contain a small number of TTX-positive granules. Above the apical surface of the epidermis, singly scattered spherical-shaped secretions (up to 3 μm in diameter) were revealed (Figure 3B).

In specimens stimulated with an electric current pulse, abundant TTX-positive secretion above the apical surface of the epidermis was revealed (Figure 3C). The epidermal ducts of subepidermal gland cells were filled by numerous TTX-positive granules (Figure 3D,D1).

## 3. Discussion

TTXs were found in representatives of 14 genera of nemerteans [2]. The spectrum of toxins of most species includes TTX and two of its equilibrium analogues (4-epiTTX and 4,9-anhydroTTX). In *Kulikovia manchenkoi*, body extracts possessed 5,6,11-trideoxyTTX, 11-deoxyTTX, and 5-deoxyTTX [8]; in *Yininemertes pratensis*, body extracts possessed 5,11-dideoxyTTX and 11-norTTX-6(S)-ol [7]. The most toxic nemertean, *C*. *simula*, contains up to 13 TTX analogues, 11 of which are non-equilibrium: 11-norTTX-6(s)-ol, 11-deoxyTTX, 11-norTTX-6(r)-ol, 5,6,11-trideoxyTTX, 5-deoxyTTX, 11-oxoTTX, 4,9-anhydro-8-epi-5,6,11-trideoxyTTX, 1-hydroxy-8-epi-5,6,11-trideoxyTTX, 4,9-anhydro-5,6,11-trideoxyTTX, 6,11-dideoxyTTX, and 4,9-anhydro-11-oxoTTX [8,11,13]. Previously, in *K*. *alborostrata* (=*Lineus alborostratus*), TTX and two of its equilibrium analogues were found [6,8]; in this study, TTX and 5,6,11-trideoxyTTX were detected. The lack of equilibrium analogues in the current research may be due to the concentration being below the method’s limit of detection (LoD).

The main supposed functions of TTXs in organisms accumulating them are defense from predators and prey capture [22], associated with the release of the toxins into the environment by specific structures. These structures include organs of the digestive system and integuments: Both types are capable of accumulating and selectively releasing TTXs [3,23,24]. Using immunohistochemistry with anti-TTX antibodies, the TTX association with specific cells in these tissues was revealed. Inside the cells, TTX was associated with the secretion-forming intracellular structures. For example, in pufferfish, the toxin is located in mucoid gland cells, sacciform cells [25,26,27], and basal cells of the epidermal layer [27]. The newts *Cynops pyrrhogaster* and *Notophthalmus viridescens* contain TTX in the glandular cells of the epidermis [28,29,30]. In venomous octopi of the genus *Hapalochlaena* (including *H. fasciata* and *H. lunulate*), TTX was found in the glandular cells lining the secretory canals of the posteriorly located salivary glands [31]. In the mollusk *Pleurobranchaea maculate*, TTX is localized in the neutral mucin cells of the mantle [23]. In nemerteans of the genus *Cephalothrix* [32] and *Micrura verrilli* [33], TTX accumulates in mucoid cells of the integumentary epithelium, and in *K*. *alborostrata* (= *L*. *alborostratus*), it accumulates in granules of type I bacillary glandular cells located in the subepidermal (cutis) layer ([21], current research). The mechanism of toxin excretion from glandular cells into the environment in response to external stimuli was described in only one work, with pufferfish [34]. The authors showed that, in response to external stimuli, TTX-containing glandular cells decreased in volume due to a contraction of the microfilaments that in turn caused an active release of the secretion. In the current study, we have shown that intact nemerteans contain a small amount of TTX-positive spherical secretion above the apical surface of the epidermis; its amount in stimulated nemerteans is appreciably higher (Figure 3). Therefore, in individuals of TTX-bearing animals that possess detectible amounts of TTX, toxin-containing glands provide a constant background of the secretion components in mucus. At the same time, they immediately respond to external stimuli with targeted secretion, which leads to the toxin concentration increasing in the surroundings.

The dynamics of toxin production by the integumentary glands were studied using in vivo models in various TTX-bearing animals by two kinds of experiments: (1) short time-interval repeated stimulation for the depletion of the toxin and (2) the subsequent recovery of the toxin during long-term captivity. In the pufferfish *Takifugu niphobles* [35], *Takifugu pardalis*, and *Takifugu poecilonotus* [36], the release of TTX-containing secretion during electric current stimulations repeated three [36] and five [35] times led to a decrease in the TTX concentration with each subsequent stimulation, down to zero. Analysis of the TTX content in the secretion of *C*. *simula* (in the article, *C*. *linearis*), produced in response to daily mechanical stimulation, showed a decrease of toxin release to amounts of <5MU on Days 3–6 [12]. The current study showed that the concentration of toxins in the majority of the *K*. *alborostrata* specimens did not recover within three days. Thus, when repeated at short time intervals, the stimulation of TTX-containing animals leads to a rapid loss of TTXs in the mucus secreted by the skin glands. The dynamic of the recovery of the toxin in the mucus to the initial concentration differed between different animal types. For example, in flatworms, TTX and its analogue 11-norTTX-6(s)-ol recovered to their initial concentrations by 8 days after secretion during hunting [37]; in pufferfish, recovery of TTX in 14 days was partial, not exceeding 77% of the initial value [35]; in newts, the rates of TTX recovery varied, and the TTX concentration recovered completely in only 2 out of 30 animals by the ninth month after secretion [38]. In the current study, in nemertean *K. alborostrata*, after 30 days since the control stimulation, the TTX level had recovered in only one-third of all 17 studied individuals, and after seven months, in 100% of individuals. The relatively rapid recovery of TTX observed in flatworms and pufferfish may indicate the use of the toxin as a repellent mucus component; however, the same function is unlikely to fit animals capable of secreting TTX only after a long recovery period, including the nemerteans studied here.

Studies of TTXs’ tissue localization in ribbon worms *Cephalothrix* sp. showed that the uptake of toxins into worms’ bodies occurs through their absorption together with food items [32], followed by toxins’ accumulation in the body wall and their subsequent migration into the skin [13]. In the current study, it was shown that, after the control stimulation during 30-day captivity without feeding, 5,6,11-trideoxyTTX completely migrated from the body wall into secreting cells and was lost during the release of secretion, which was supported by the fact that, after 7 months, it was discovered neither in the secretion nor in the body. Therefore, taking into account the toxin regeneration in the secreting structures of the studied *K. alborostrata* nemerteans, it can be assumed that 5,6,11-trideoxyTTX, due to its relatively rapid recovery in secretions, can be purposefully used by nemerteans. However, the study of 5,6,11-trideoxyTTX interaction with the Nav channels investigated in mammals showed its low affinity for channels [39,40]. Thus, to assess the effect of this compound as a repellent agent, further studies of the interaction of this TTX analogue with the Nav channels of organisms, whose diet may include nemerteans, are required.

In some TTX-bearing animals, a mechanism of TTX resistance was found, consisting of amino acid substitutions in the Nav channels’ positions contributing to TTX coordination and binding. Data indicating the existence of these substitutions in selective filter Nav channel regions in some animals have been published in a number of studies, and their probable relationship with the ability to accumulate TTX has been noted. These animals included many species of invertebrates, comprising mollusks, jellyfish, flatworms, some insects, and arachnids [41,42,43], as well as vertebrates, such as newts, snakes [44,45,46], and the most famous TTX-containing animal, pufferfish [47,48]. Electrophysiological studies were carried out to examine the effect of founded substitutions on the Nav channels’ affinity for TTX [16,18,46]. In a recent study, the structure of the Nav channel TTX binding site was described, and a model of the detailed coordination of TTX by amino acid residues of the Nav channel pore of the cockroach *Periplaneta americana* was constructed, according to which TTX blocks the access of Na^+^ ions to the vestibule of the selective filter through the formation of electrostatic interactions with amino acids in the outer electronegative ring, as well as Asp and glutamic acid in the DEKA motif [17]. In the current study, the Nav1 channels of all 17 studied specimens of *K. alborostrata* possess identical amino acid substitutions that may contribute to its resistance to TTX according to the literature [40,41,42,43,44].

## 4. Materials and Methods

**Figure 4:**
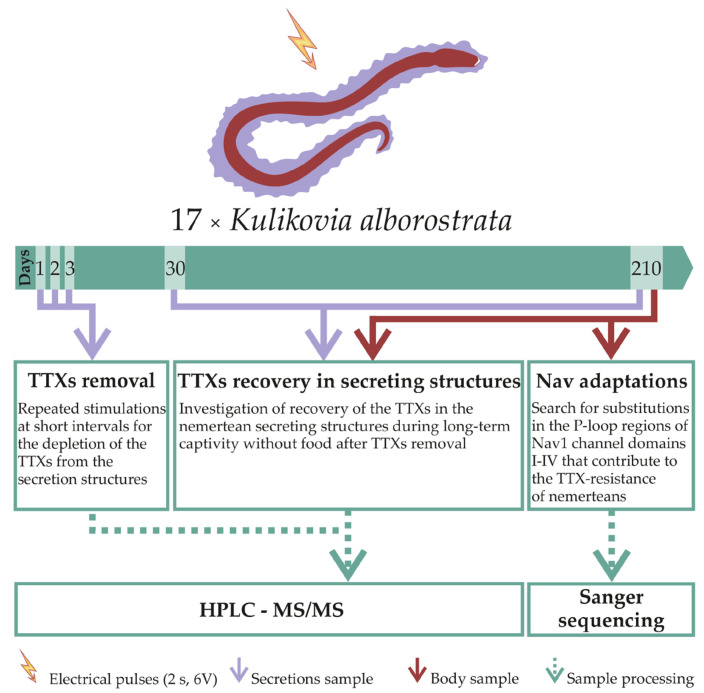
Scheme of the experimental design for studies of tetrodotoxins (TTXs) secretion and Nav1 adaptation in the ribbon worm *Kulikovia alborostrata*.

In July 2020, samples of *K*. *alborostrata* (Takakura, 1898) ribbon worms were collected in Spokoinaya Bay (42.7090N, 133.1809E) in Peter the Great Gulf (42.7090N, 133.1809E) (Sea of Japan) at a depth of 0.5 to 2 m. The identification of species was kindly carried out by Prof. Chernyshev, a specialist in the zoology of Nemertea. Live individuals were kept in aquaria with running seawater (t = 17 °C). For the TTX localization study, 4 specimens were taken: 2 specimens were stimulated with an electric current pulse (stimulated animals), while the other 2 specimens were not stimulated (intact animals). Next, the worms were anesthetized with 7% MgCl_2_ and processed for subsequent immunohistochemistry studies (Section 4.3). To study the TTXs secretion (Figure 4), 17 individuals were kept in aerated aquaria with filtered seawater (t = 12 °C) for 210 days without feeding; the water in the aquaria was changed once a week. Then, the animals were stimulated to produce secretions. On the first, second, and third days; after 30 days; and after 210 days of keeping, the secretion was used for the subsequent isolation of TTXs (Section 4.1). After the last secretion collection, the ribbon worm body was divided into parts, and a 10-mg fragment was prepared for RNA isolation (Section 4.2.2); the remaining body was used for the subsequent isolation of TTXs (Figure 4). Body and secretion extracts were analyzed by HPLC-MS/MS (HPLC-MS/MS analysis was conducted in School of Biomedicine of Far Eastern Federal University) (Section 4.1).

### 4.1. TTXs

To collect secretions, ribbon worms were placed individually on a disposable Petri dish with 1 mL of sterile seawater and were stimulated by a short electric current pulse (2 s, 6 volts) by placing copper electrodes in the water. The secreted mucus was collected together with water in 15-milliliter test tubes. Care was taken to ensure that all animals remained active after stimulation. Secretion and body samples were frozen at −20 °C for further TTX isolation and analysis by HPLC-MS/MS.

TTX isolation and analysis by HPLC-MS/MS were carried out according to the procedure of Vlasenko and Magarlamov [5]. The recovery rate of the method was 83.6%. A calibration curve for TTX concentration determination was built with a series of TTX standard dilutions (Alomone Labs Ltd., Jerusalem, Israel). The parameters for the detection of TTX and its analogues, including multiple reaction monitoring (MRM) transition peak S/N ratio of >3, toxins elution order, and the relative intensity of the fragment ion peak of >4%, corresponded to those described in Bane et al. [45]. The MRM transitions used for TTX detection corresponded to those described in the works of Vale [46], Kudo et al. [47], Bane et al. [3], Puilingi et al. [48], and Turner et al. [49]. The identification of 5,6,11-trideoxyTTX was carried out using the toxin standard isolated from *C*. *simula* as previously described [5]. The calculation of TTX analogues’ concentrations was performed by following the procedure described by Chen et al. [50]. The method was validated using standard TTX solutions in MRM mode. The linearity range was from 0.6 to 100 ng/mL. The recovery range from 1 to 100 ng/mL of TTX was 98.4%. The LoQ was 0.6 ng/mL; the LoD was 0.2 ng/mL, and the relative standard deviation was 4.5–14.6%.

### 4.2. Nav1 Channel

#### 4.2.1. Design of Primers for the Amplification of P-Loop Regions of the Nav1 Channel Domains I–IV

The design of primers was based on the reference Nav1 channel sequence from the annotated transcriptome of the *Notospermus geniculatus* heteronemertean [49]. The 5524-base-pair transcript TRINITY_DN170343_c0_g1_i6 was chosen, which was confirmed as belonging to the Nav1 family by the amino acid configuration of the selective filter (DEKA). Due to the lack of information on the Nav1 of other nemerteans, a search for Nav1 channels of heteronemerteans and the design of primers for their amplification was performed using transcriptomes deposited in the SRA (Sequence Read Archive) NCBI database (https://www.ncbi.nlm.nih.gov/sra, accessed on 30 May 2021). A reference transcript of *N. geniculatus* was preliminarily manually completed to a length of 6339 bp using a read search in the SRA database. For further work, short reads were taken from the transcriptomes of *Lineus sanguineus* (SRR3581123, SRR3581122, SRR3581110, SRR3581119), *Lineus ruber* (SRR3581105, SRR1324988, SRR1324987), and *Lineus longissimus* (SRR3938996, SRR1324985, SRR1324986, SRR2682192). A read quality assessment was carried out using the FastQC v0.11.7 software package (https://www.bioinformatics.babraham.ac.uk/projects/fastqc/, accessed on 30 May 2021). The trimming of adapters and low-quality sequences (Q-score < 20) was performed with Trimmomatic v0.27 [50]. After preliminary processing, the obtained reads were aligned to the reference Nav1 channel of *N. geniculatus* using the BOWTIE2 v2.4.2 software package [51]. Selected reads were assembled into longer contigs using Geneious prime 4.3.8 (https://www.geneious.com, accessed on 30 May 2021). The conserved regions were determined by the obtained contigs and *N. geniculatus* transcript alignment using MEGA7 [52]. Primers for conserved regions flanking the selective filter of the Nav1 channel were designed using Primer Premier 5 (http://www.premierbiosoft.com/primerdesign/, accessed on 30 May 2021) (Table 2).

#### 4.2.2. The Isolation of RNA, Synthesis of cDNA, and Amplification of P-Loop Regions of Domains I–IV of the Nav1 Channel

The isolation of the RNA of the ribbon worm *K. alborostrata* was performed using TRIzol Reagent (Thermo Fisher Scientific, Waltham, MA, USA) in accordance with the manufacturer’s protocol. The RNA concentration and quality were assessed using a BioPhotometer nucleic acid analyzer (Eppendorf, Hamburg, Germany). The length of the isolated fragments was assessed by agarose gel electrophoresis. Double-stranded cDNA was synthesized using the MINT2 kit (Evrogen, Moscow, Russia) in accordance with the manufacturer’s protocol. Double-stranded cDNA was isolated from the reaction mixture using a QIAquick PCR Purification Kit (QIAGEN, Hilden, Germany). The concentration and quality of the isolated cDNA were assessed using a BioPhotometer instrument. Next, PCR amplification of the selective filter regions of domains I–IV of the Nav1 channel gene was performed using the Tersus kit (Evrogen). The amplification working program included the following stages: (1) the initial heating of the mixture at 94 °C for 2 min; (2) 33 cycles of denaturation at 94 °C for 30 s, primer annealing for 25 s at the temperatures indicated in Table 2 for each pair of primers, and elongation at 72 °C for 40 s; and (3) the final extension of DNA strands at 72 °C for 5 min. The presence of target amplicons was assessed by gel electrophoresis. Isolation of amplicons from the gel was performed using a QIAquick Gel Extraction kit (QIAGEN), and the concentration and quality of the extracted DNA were assessed using a BioPhotometer device.

#### 4.2.3. Sanger Sequencing

Determination of the nucleotide sequence of the amplified *K. alborostrata* Nav1 channel fragments was performed by Sanger sequencing using Brilliant Dye (Nimagen, Nijmegen, Netherlands). The amplification working program included the following steps: (1) the initial heating of the mixture at 96 °C for 1 min; (2) 28 cycles of denaturation at 96 °C for 10 s, primer annealing at 50 °C for 5 s, and elongation at 60 °C for 4 min; and (3) the final extension of DNA strands at 60 °C for 4 min. The PCR product was precipitated and then washed and redissolved in formamide. Capillary electrophoresis was performed in a Genetic Analyzer 3500 (Applied Biosystems, Foster City, CA, USA).

#### 4.2.4. Search for Mutations in the P-Loop Regions of Domains I–IV of the Nav1 Channel

The search for amino acid substitutions was carried out by aligning the P-loop regions of *K. alborostrata* Nav1 domains I–IV obtained by sequencing with the protein sequences from the Uniprot and NCBI databases of Nav channels in human *Homo sapiens* Nav1.4 (P35499), pufferfish *T. rubripes* Nav1.4b (Q2XVR6), *T. rubripes* Nav1.6b [16], newt *Taricha granulosa* Nav1.3 (A0A6G9W273), and garter snake *Thamnophis sirtalis* Nav1.4 (A0A1W5T2B2), using the multiple alignment algorithm ClustalW in MEGA7.

### 4.3. Immunohistochemical Study

For the confocal laser scanning microscopy (CLSM) analysis, body fragments from the anterior intestine region of the adult animals were fixed for 1.5 h at room temperature with 4% paraformaldehyde in phosphate-buffered saline (PBS, pH 7.4). The samples were rinsed with PBS and incubated in a 20% sucrose solution in PBS for 1 h at + 4 °C. After that, the samples were placed in a Leica OCT cryocompound tissue-freezing medium solution (Leica, Wetzlar, Germany), and transverse sections (about 10-μm thick) were made with a Thermo HM 560 microtome (Thermo Fisher Scientific) and transferred to slides (Thermo Fisher Scientific). Sections were permeabilized for 1 h in 1% Triton-x100 in PBS, rinsed in PBS, and incubated overnight in blocking solution (5% bovine serum albumin in PBS) at +4 °C. For TTX detection, the primary antibodies were dissolved in PBS (Genetex, Irvine, CA, USA, 1:25); for visualizing the tubulin-like immunoreactivity structures, anti-acetylated tubulin primary antibodies were dissolved in PBS (Santa Cruz, CA, USA,1:2000). The material was kept in the primary antibody at +4 °C for 2 d, then rinsed in PBS and incubated for 24 h at +4 °C in a mixture of the secondary antibodies Alexa 488 goat anti-mouse (Invitrogen, Waltham, MA, USA, diluted 1:500) and Alexa 647 goat anti-rabbit (Invitrogen, USA, diluted 1:500) in PBS. Then, the sections were washed in PBS, immersed in Mowiol 4–88 (Sigma–Aldrich, Burlington, MA, USA) and analyzed on an LSM-780 microscope (Carl Zeiss, Jena, Germany). The obtained image series were analyzed with the CLSM-780 software, and the images were further processed with Photoshop CS2 to adjust the contrast and brightness and to create digital line drawings.

## 5. Conclusions

In the current study, we carried out experiments to investigate the processes and adaptations aimed at the accumulation and usage of TTX and 5,6,11-trideoxyTTX by nemertean *K. alborostrata*. The study included (1) an analysis of the dynamics of toxins’ concentrations in the secretion produced in response to irritation during repeated stimulations at short intervals, (2) quantification of toxins’ concentrations in ribbon worms after prolonged captivity without food, (3) study of the migration pathways of toxins during excretion, and (4) a search for mutations in the genes of Nav1 channels that contribute to the TTX resistance of nemerteans. The studies have shown a low rate of TTX recovery in the nemertean secreting structures; after 30 days, TTX was recovered in only one-third of the studied ribbon worms. This leads us to suggest a low probability of targeted usage of TTX by the toxic nemertean *K. alborostrata* as a repellent through its secretion in mucus. The TTX analogue 5,6,11-trideoxyTTX, on the contrary, fully migrated from the body wall into the secreting cells and was completely lost through the release of secretion after 30 days of the experiment, which may indicate the specificity of its transfer in response to stimulation and possible targeted usage. The current study revealed that the sequences of the P-loop regions of Nav1 channel domains I–IV of all 17 studied specimens are identical and have amino acid substitutions, which were shown for TTX-resistant organisms, and, according to the literature data, can contribute to TTX resistance.

## Figures and Tables

**Table 1:** Tetrodotoxin (TTX) and 5,6,11-trideoxyTTX in extracts of nemertean *Kulikovia alborostrata*.

№. of Specimen			1	2	3	4	5	6	7	8	9	10	11	12	13	14	15	16	17
Secretion (TTXs, ng/g)	Day 1	TTX	+	+	+	-	+	-	+	-	-	-	-	-	-	-	-	-	-
		5,6,11-trideoxyTTX	0.675	0.264	0.229	-	-	-	-	-	-	-	-	-	0.144	-	-	-	-
	Day 2	TTX	0.340	0.357	+	0.556	+	+	+	+	+	-	+	+	-	-	-	-	+
		5,6,11-trideoxyTTX	1.905	3.171	0.156	6.244	0.399	-	1.690	0.774	-	0.440	-	0.835	-	-	-	-	0.199
	Day 3	TTX	-	-	-	-	-	-	-	+	-	-	-	-	-	-	+	-	-
		5,6,11-trideoxyTTX	-	-	-	-	-	-	-	-	-	-	-	0.287	0.115	-	0.093	-	-
	Day 30	TTX	+	+	-	0.306	-	-	-	-	-	+	-	+	+	-	+	-	-
		5,6,11-trideoxyTTX	-	+	+	-	0.338	+	0.862	0.413	+	0.253	0.178	0.547	0.140	+	0.068	-	+
	Day 210	TTX	+	+	+	+	+	+	+	+	0.305	+	+	+	+	+	+	+	+
		5,6,11-trideoxyTTX	-	-	-	-	-	-	-	-	-	-	-	-	-	-	-	-	-
Body (TTXs, ng/g)		TTX	+	+	+	1.166	0.492	0.146	+	+	1.286	+	0.306	0.441	+	+	+	+	+
		5,6,11-trideoxyTTX	-	-	-	-	-	-	-	-	-	-	-	-	-	-	-	-	-

+: <limit of quantification (0.6 ng/mL of extract); -: not detected.

**Table 2:** Primers for the amplification of the P-loop regions of domains I–IV of the *Kulikovia alborostrata* Nav1 channel.

Primer	Sequence	T Annealing, °C	PCR Product Length, bp
DI forward	ATGCGCCTTTCGCCTTATGAC	61.7	233
DI reverse	CGGCGTTCTTCCTCTTCCTTT	60.6	
DII forward	GTCCT(Y)CGAACATTCAGATTG	61.1	431
DII reverse	AGATTGGAGATTTTCAGCCCC	59.9	
DIII forward	GTCTTCTGGCTCATCTTCAGTATCA	59.8	348
DIII reverse	TCAGCGTGAAGAAAGAACCGA	60.9	
DIV forward	AACATGCTGCCGGGATAGA	58.8	193
DIV reverse	TTGCCGCAGTTACCCTTGAC	60.9	

## Data Availability

The Nav1 partial gene sequences of *Kulikovia alborostrata* and *Notospermus geniculatus* have been deposited in GenBank under the accession numbers: MZ508867, MZ508868, MZ508869, MZ508870 and MZ508871.
